# Challenges and possibilities of enabling person-centred engagement in medication safety with older people living at home

**DOI:** 10.1093/intqhc/mzaf122

**Published:** 2025-12-16

**Authors:** Elizabeth Manias, Chris Moran, Jacqueline Allen, Stephanie Garratt, Roisin McNaney, Helen Rawson

**Affiliations:** Monash Nursing and Midwifery, Monash University, Clayton 3800, Australia; School of Public Health and Preventive Medicine, Monash University, Melbourne 3004, Australia; Department of Aged Care, Alfred Health, Melbourne 3004, Australia; Monash Nursing and Midwifery, Monash University, Clayton 3800, Australia; Monash Nursing and Midwifery, Monash University, Clayton 3800, Australia; School of Computing and Information Systems, The University of Melbourne, Parkville 3052, Australia; Monash Nursing and Midwifery, Monash University, Clayton 3800, Australia

## Background

Medication safety is a complex and continuous process for older people where potential medication-related harm can occur with starting, taking, adding, reviewing, and stopping medications. Preventable medication-related harm relating to individual or system failures, occurs at a rate of 5% of people globally. Communication breakdowns are a major cause of preventable medication-related harm for older people living at home [1]. In addressing communication breakdowns, person-centred engagement is advocated. Despite years of person-centred promotion for engagement, actual practice has often failed to demonstrate shifts in improving uptake of these initiatives [1]. The aim of this perspectives paper is to examine challenges and identify possibilities for person-centred engagement with older people during communication encounters around medication safety.

## Communication between health professionals with older people

As people age, they often develop multiple comorbidities requiring medication management, which occurs through a complex web of healthcare consultations with general practitioners, medical specialists, community nurses, pharmacists, and allied health workers. By their nature, consultations tend to focus on diagnosing and treating the patient’s medical complaint. Communication about medications tends to be about how and whether older people follow their recommended medication regimen and the need for further supplies, and while there is consideration of older people’s views about their medications, challenges exist [2].

Healthcare consultations often comprise facilitating adherence and improving medication knowledge without communicating about how to anticipate and respond to possible concerns with medications [2]. Barriers to communication commonly occur relating to implicit bias. Implicit bias is where health professionals make assumptions about what they expect older people to know, which may limit possibilities in decision-making about their medication needs. Such situations could perpetuate lack of confidence, medical paternalism, and fear of speaking up [2].

To address challenges in communication between health professionals and older people, an expansive array of resources has been developed globally. The Health Innovation Network in England has information tools to support people to understand key issues in managing their medications, including the questions they may want to ask. These resources are available in multiple languages with easy-read versions for those of low health literacy [3]. Similarly, the Canadian Deprescribing and Medication Appropriateness Network has resources to help people in conversing about medication safety and deprescribing [4].

A randomised controlled trial about the Choosing Wisely questions, which are five questions that people can ask doctors about their treatment, showed that this resource did not produce significant improvements in question-asking and decision-making outcomes based on participants’ health literacy. Compared with those with adequate health literacy, people with limited health literacy were less likely to have positive attitudes towards shared decision-making, identified they would follow low-value treatment plans without further questioning, and were less likely to ask about treatment risks, costs and alternative measures [5]. It is possible for inequities to be pronounced, where individuals who are struggling the most, are less likely to ask questions about their medications. Thus, the mere availability of questions is insufficient to promote involvement in decision-making.

## Targeted communication about medication-taking activities

Aside from healthcare consultations, targeted medication reviews are undertaken in an effort to support older people in managing their medications. Medication reviews may be undertaken in primary care clinics or in older people’s homes, which incorporate comprehensive medication assessment to identify safe and effective use. This assessment includes health professionals’ focus on optimising prescribing and attempting to promote deprescribing.

Assessment includes efforts to reduce or stop medications, as advocated in guidelines [3, 4], but these are sensitive matters to be negotiated, especially in situations where older people have taken these medications for prolonged periods. While evidence shows that older people are willing to accept deprescribing compared to their younger counterparts [6], complexities exist. Older people may decline to deprescribe even with shared decision-making with their general practitioners, and while they may agree to having any of their medications stopped, this view has not been associated with reductions in medication prescribing over time [7]. Such negotiations are very delicate and require careful consideration between health professionals and older people in terms of how decisions are made to achieve the desired outcome. In a quality improvement project, resources produced by the Health Innovation Network showed 66% of people at risk of polypharmacy and living in high deprivation areas stated the resources helped them to share what they viewed was important, and 58% indicated the resources supported them in preparing for a structured medication review. Challenges identified were if they received the resources at all or in a timely way, and their lack of understanding about the role of pharmacists in medication reviews. There were also concerns about sustainability and whether health professionals were likely to continue to use the resources in their day-to-day practice [8].

## Creating possibilities for person-centred engagement

Creating possibilities for person-centred engagement with older people living at home requires a multi-faceted approach that recognises the individuals’ uniqueness and value through an equity lens. Guidelines are available to improve shared decision-making about medications using a person-centred approach [9, 10]. This approach involves health professionals taking proactive action to negotiate an agreed-upon agenda with older people about their medication regimen and to elicit concerns at the beginning of conversations, which shape future discussion [2, 3]. Some older people wish to include families in decision-making, and if this is the case, health professionals need to incorporate family perspectives in medication conversations. Inviting older people to show and talk about their medications can help to prioritise goals, to prompt questions that relate to their situation, and to appraise the role of medications in their daily lives by reflecting on their values, preferences, and needs. Also important is the role of staff training to ensure health professionals have the knowledge, skills and confidence in promoting person-centred engagement and shared decision-making about medications [10]. The creation of safe spaces in medication conversations needs to be an iterative process that builds on each encounter to instil confidence, respect and trust in older people and their families, and the belief that their contributions really matter.

Conflict of interest: None declared.
